# A Poultry Value Chain Intervention Promoting Diversified Diets Has Limited Impact on Maternal and Child Diet Adequacy during the Lean Season in a Cluster Randomized Controlled Trial

**DOI:** 10.1093/jn/nxac034

**Published:** 2022-02-16

**Authors:** Elodie Becquey, Loty Diop, Josue Awonon, Ampa D Diatta, Rasmane Ganaba, Abdoulaye Pedehombga, Aulo Gelli

**Affiliations:** International Food Policy Research Institute (IFPRI), Washington, DC, USA; International Food Policy Research Institute (IFPRI), Washington, DC, USA; International Food Policy Research Institute (IFPRI), Washington, DC, USA; International Food Policy Research Institute (IFPRI), Washington, DC, USA; AFRICSante, Bobo-Dioulasso, Burkina Faso; AFRICSante, Bobo-Dioulasso, Burkina Faso; International Food Policy Research Institute (IFPRI), Washington, DC, USA

**Keywords:** behavior change communication, cluster randomized controlled trial, dietary diversity, micronutrient intake, nutrition-sensitive poultry value chain

## Abstract

**Background:**

Soutenir l'Exploitation Familiale pour Lancer l’Élevage des Volailles et Valoriser l’Économie Rurale (SELEVER) is a nutrition- and gender-sensitive poultry value chain project designed and implemented by international nongovernmental organization Tanager, which consists of poultry market facilitation and behavior change activities aiming at increasing poultry production and improving diets without free inputs transfer.

**Objectives:**

The study aimed at assessing the impact of SELEVER on diets of women and children during the lean season.

**Methods:**

Within a cluster randomized controlled trial, 45 communes were assigned to 1 of 3 arms, including *1*) SELEVER interventions, *2*) SELEVER with an intensive hygiene and sanitation component (SELEVER + WASH), and *3*) a control group without intervention. Two rounds of survey were conducted 2 y apart during the lean season. Primary dietary outcomes were the probability of adequacy (PA) of iron, zinc, and vitamin A intakes; mean PA of 11 micronutrients and individual dietary diversity score collected through quantitative 24-h recall in longitudinal samples of women and index children (2–4 y old) in 1054 households; and minimum acceptable diet in the repeated cross-sectional sample of their younger sibling aged 6–23 mo. Impacts were assessed by intention-to-treat ANCOVA.

**Results:**

Relative to control, SELEVER interventions (groups 1 + 2) increased the PA of iron intakes in women by 1.8 percentage points (pp) (*P* = 0.030). We found no further impact on primary outcomes, although egg consumption increased in index children (+0.73 pp, *P* = 0.010; +0.69 kcal/d, *P* = 0.036). Across the 3 groups, we observed negative effects of SELEVER on the PA of zinc intakes in women relative to SELEVER + WASH (–4.1 pp, *P* = 0.038) and on a variety of secondary dietary outcomes relative to both other groups. The study was registered on the ISCRCTN registry (ISRCTN16686478).

**Conclusions:**

Information-only-based value chain interventions may not have meaningful positive effects on diets of women and children in the lean season in settings with largely inadequate diets. We found suggestive evidence that synergies between intervention components may have introduced heterogeneity in effects on diet.

## Introduction

Recent estimates on the global burden of disease attribute 20% of deaths to unhealthy diets (1). In their development of the United Nations 2030 Sustainable Development Agenda, policymakers highlighted the need for agricultural programs to support improved diets, nutrition, and health. Nutrition-sensitive programs can be leveraged to deliver nutrition interventions at scale (2). In particular, evidence reviews have found that integrated agriculture and nutrition interventions consistently improve household access to nutritious foods, leading to improvements in the diets of mothers and young children (2, 3).

Within nutrition-sensitive agriculture intervention, livestock interventions in particular can provide low-income households with both a livelihood and a source of high-quality protein and bioavailable micronutrients (4, 5). Poultry interventions are particularly relevant for poverty alleviation due to their near ubiquity in low-income settings (6), the potential market opportunity from the demand from urban consumers to accelerate poultry-sector transformation, the relatively modest investment needed, and the potential contribution from eggs and poultry meat to diets in both rural and urban settings (7).

Despite this potential, there is little rigorous evidence on the role of livestock interventions in improving diets, particularly those involving information only (3). The potential role of interventions in food value chains in improving diets has received recent attention, including the need to consider how food is produced, processed, distributed, and marketed (8–10). However, there is also a dearth in the evidence on the effectiveness on diet outcomes of scaling up nutrition interventions through value chain and market facilitation platforms. The 1 experimental study we are aware of that measured diet outcomes in the context of a poultry value chain intervention found promising results in Ethiopia, with a positive impact of the intervention on child diet diversity (11), although the impact on the micronutrient adequacy of the diet was not assessed and remains unknown. Yet, the positive impact on diet diversity was not found in the lean season. However, in countries such as Burkina Faso (our country of focus), evidence shows that overall diet adequacy significantly decreases in the lean season, when food insecurity increases (12–14). Therefore, the burning question of the actual effectiveness on diet adequacy of poultry value chain interventions aiming at improving diets needs to be answered while considering the possible modifying effect of the season.

This study aimed at providing new experimental evidence on the impact on the diets of women and young children during the lean season of an integrated livestock production and nutrition intervention implemented in a poultry market system in Burkina Faso. Our hypothesis was that impact estimates during the lean season would be lower bounds for effectiveness of behavior change communication (BCC)–type interventions because rural households face higher resource constraints and consume lower-quality diets compared with the postharvest period. This article draws on the prespecified analysis of data from a subsample of the Soutenir l'Exploitation Familiale pour Lancer l’Élevage des Volailles et Valoriser l’Économie Rurale (SELEVER) trial and focuses on the diet-related primary outcomes of the trial (15). The results on the other primary outcomes (poultry production and marketing) in the lean season have been published separately (16).

## Methods

### Country context

Burkina Faso, a Sahelian country, chronically suffers from high rates of child and maternal malnutrition (17). Infant and young child feeding (IYCF) practices are particularly poor. A recent study estimated that Burkina Faso had the second lowest dietary diversity score in the world (18). Fourteen percent of children younger than 2 y had consumed poultry flesh, and egg consumption was limited to 3% of children in the same age group, whereas 80% of households owned poultry.

### Intervention

SELEVER, or the Women's Poultry Program to Improve Income and Nutrition project, funded by the Bill & Melinda Gates Foundation, was designed and implemented by the international nongovernmental organization (NGO) Tanager in partnership with in-country NGOs, private institutions, and government services. SELEVER aimed at increasing poultry production and improving the diets and nutritional status of women and children. The project approach involved a set of components combining poultry revenue generation, women's empowerment, and nutrition BCC, and it specifically excluded any input or food distribution for free. The roll-out at the community level by the NGOs involved cascade trainings (i.e., training of trainers), follow-up home visits, peer-group support, and advocacy/sensitization, and it was facilitated and/or conducted by key community members such as religious or traditional community leaders, women leaders, “champion husbands,” and “model women.”

The poultry component included training of volunteers on poultry husbandry and of village volunteer popularizers to improve the quality of their extension services. Trainings included nutrition-related modules for the promotion of consumption of animal source foods and basic hygiene practices, as well as messaging on women's empowerment. Other activities at the community level included leveraging micro-credit groups as platforms to implement the poultry-related trainings.

The nutrition component included BCC on nutrition and diets provided through women's groups, poultry producer groups, and local community leaders. The topics of the BCC activities included basic hygiene and the promotion of improved diets at key stages of the life cycle: this included IYCF practices and diet diversity promotion through the promotion of daily consumption of 3 key food groups: energy-giving foods (starchy staples and fats), protective foods (fruit and vegetables), and body-building foods (animal source foods, legumes, and nuts). The gender component included community-level sensitization on women's economic empowerment and gender equity, including strengthening of women's groups, training participants from existing women's associations on enterprise development, and strengthening women's role in decision-making within households and in the community.

The program impact pathways through which the integrated agriculture and nutrition intervention could affect children's diets were based on the program theory of value chain for nutrition interventions (19). Briefly, the SELEVER package could have an impact on diets through 4 interlinked pathways based on *1*) leveraging demand, *2*) supplying nutritious foods, *3*) enhancing nutrition-related value addition along a chain, and *4*) empowering women (15).

### Study design

A cluster randomized controlled trial was designed to assess the 3-y impact on dietary and poultry production outcomes of the SELEVER intervention, with (SELEVER + WASH) or without (standard SELEVER) additional poultry-related hygiene and sanitation (WASH) behavior change activities inspired from the community-led total sanitation approach. This article presents an intermediary analysis to assess the impact of the intervention on dietary outcomes (see below) during the lean season after 2 y of implementation. The overall study protocol was published elsewhere (15).

### Sampling design

**FIGURE 1:**
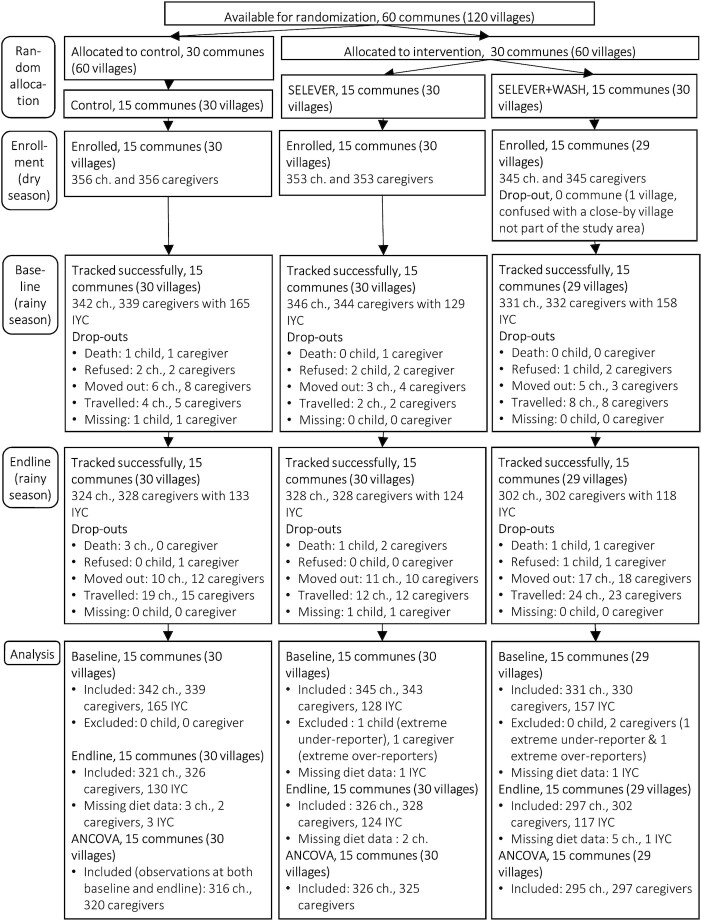
Flowchart of study participants, including longitudinal samples of index children (ch.) and caregivers and cross-sectional sample of infants and young children (IYC). SELEVER, Soutenir l'Exploitation Familiale pour Lancer l’Élevage des Volailles et Valoriser l’Économie Rurale.

The study area includes rural and periurban communities from 60 communes within the Hauts-Bassins, Boucle du Mouhoun, and Centre-Ouest regions. The random allocation was undertaken in 2 stages through restricted randomization by modeling selection using a set of commune- and village-level variables obtained from the 2006 census (20). During the first stage of randomization, communes were randomly assigned to 1 of 2 treatment arms (SELEVER treatment compared with control). The second stage of randomization further divided the treatment communes into 2, including a SELEVER group (standard SELEVER intervention) and a SELEVER + WASH group with additional poultry-WASH BCC activities (**Figure 1**). The control communes were also further divided into 2, and diet data collection was conducted in 15 control communes. The program randomly selected 2 villages in each commune. In each village, 12 households with children in the 2- to 4-y age group were randomly selected from a census conducted prior to the baseline survey, with overrepresentation of large poultry-producing households (defined as owning >20 chickens/fowls at baseline), and an index child in the 2- to 4-y age range was then randomly selected for inclusion in the biomedical component of the analysis, with the primary female caregiver. We also surveyed repeated cross-sectional samples of infants and young children (IYC sample) comprising, for each survey round, the youngest child aged <24 mo of the index caregiver, if any. The power calculations for selecting sample size for women and target children were based on 80% statistical power and α = 0.05, and they were calculated using data from an observational study examining food intake in 2 of the 3 selected regions (12, 21).

### Primary outcomes

Primary outcomes for women (aged 15–49 y at baseline) and target children (aged 2–4 y at baseline) were Individual Dietary Diversity Score (IDDS, defined as the number of food groups consumed the previous day of 10 standard food groups) (22), and the probability of adequacy (PA) of intake for iron, zinc, and vitamin A, as well as mean probability of adequacy (MPA) in micronutrient intake of 11 micronutrients. Minimum acceptable diet in children aged 6–23 mo (23) was defined as the primary outcome in the cross-sectional IYC samples. Household poultry production, sales, and profits were the remaining primary outcomes of this study and were published separately (16).

### Data collection

Data collection was performed electronically using a user-friendly computer-assisted personal interview survey form designed in the application SurveyBe. Enumerators completed the survey on Android and Microsoft Windows tablets. All survey tools were written in French, and the enumerators spoke both French and local languages.

Enumerators visited the household a first time and collected a wide range of indicators at village, household, caregiver, and child levels. Index women and their husbands were separately asked about their participation in the various activities related to SELEVER over the previous 12 mo. Individual responses were aggregated to obtain household-level estimates of exposure. Standard IYCF practices were then collected through recall with caregivers of children in the IYC sample (23). At the end of this visit, enumerators distributed standard kitchen utensils commonly used in the area. They instructed women to not change their eating habits or the ones of the index child on the following days, except for both eating separately from the rest of the family and from each other, using 1 plate and 1 bowl each to serve their food. This was intended to minimize difficulties in quantifying individual dietary intakes, as serving food in a common pot was standard practice. Enumerators also emphasized that the mother paid attention to quantities consumed for herself and her child. Although such recommendations might slightly influence behavior, we expected gains in accuracy of data and did not expect this potential bias to differ by treatment group.

Two or 3 days after the first visit, dietary intake data were collected by specifically trained enumerators using an interactive 24-h recall method (24). A second recall was collected at least 2 d after the first recall in 2 randomly selected dyads per village. All days of the week were considered. The steps in the recall were as follows:

- First pass, caregiver: the respondent recalled the complete list of all foods, drinks, and snacks consumed during the previous 24-h period.
- Second pass, caregiver: a precise description and mode of preparation of all foods consumed, including recipes for mixed dishes, allowed the enumerator to select the appropriate foods within a preloaded, comprehensive list of foods based on previous work in Burkina Faso (25, 26) and the FAO food Composition Table for West Africa (27).
- Third pass, caregiver: the respondent was prompted to mentally visualize and then quantify the amount of each ingredient used in recipes, as well as the size of the portions consumed, using the most appropriate method (see below). Wasted and nonconsumed parts of foods and ingredients were documented.
- Passes 1–3, child: Once the recall was finished for the mother, the enumerator used the same method to recall food consumption of her child.
- Fourth pass: The enumerator recapitulated the whole list of foods consumed by both the mother and her child to verify if every food was correctly listed and quantified for each respondent.

Prior to the survey, each unique food in the food list had been assigned a preferred measurement method and defined other authorized measurement methods. Enumerators were made aware of these methods through the software. These methods included the weighing of a replicate, volume measurements with water, referring to a picture atlas, modeling food size with clay or using wooden or plastic models, calibrating household measures, or collecting prices.

### Data management and indicators creation

All data management was executed in Stata (StataCorp LLC). Volumes and household measures were converted to weight values using conversion tables of density and of specific household measures. Conversion factors were calculated as the average of household-specific conversion factors collected in other households of the whole sample, when available, or were collected through a separate market survey, and some came from a previous survey (26).

Food composition table (FCT) and the table of edible proportions were based on the FAO FCT and previous literature and published work in Burkina Faso (25, 27, 28). To account for nutrients lost during cooking, retention factors were applied to all foods that underwent heat exposure during preparation (29).

Classification of foods into 10 food groups followed the FAO's Minimum Dietary Diversity for Women guidelines for index children and caregivers (22). Unclassified foods included spices, sugar, salt, oils, and other condiments, defined as foods consumed in quantities of <10 g in the day (30). For the IYC samples, we compiled IYCF indicators according to standard 2007 guidelines (23).

We used the National Cancer Institute approach to calculate distributions of usual intakes of 11 micronutrients (vitamin A, vitamin C, thiamin, riboflavin, niacin, vitamin B-6, vitamin B-12, folate, calcium, zinc, and iron) (31). Then, we used the probability approach to calculate the PA of intake for these 11 micronutrients (32). We used the relevant estimated average requirements and standard deviations for age, sex, and physiologic status of the European Food Safety Authority's dietary reference values for nutrients (33). We adjusted requirements assuming low levels of bioavailability for iron (5%) and zinc (15%) due to high cereal consumption and low animal product consumption in our population. The MPA was calculated as the mean of the 11 micronutrient PAs.

### Data analysis

We followed an intention-to-treat approach and used a single-difference ANCOVA controlling for village-level clustering (using robust estimations of standard errors) and taking sampling weights into account to examine effects in first-level (treatment compared with control) and second-level (SELEVER + WASH compared with standard SELEVER) comparisons. For ease of interpretation of coefficients, the regressions used linear (probability) models for both continuous and binary variables. In the latter case, if robust estimations of standard errors are computed, these models produce valid coefficients that represent percentage point (pp) changes in probability (34). All analyses adjusted for the baseline value of the outcome, as well as for age and gender (children's outcomes) or for age and physiologic status (caregivers’ outcomes). Analyses on diet outcomes were further adjusted on whether a market occurred the previous days as this can influence consumption (35). The level of significance was set at 5%. We also discussed robustness of results of the 3 study group comparisons using a level of significance of 1.7%, which adjusts for multiple testing using the Bonferroni method. Statistical analyses were conducted using Stata 16.0 (StataCorp LLC).

### Ethics

Ethical clearance was obtained from the Comite d’éthique pour la Recherche en Santé MS/MRSI in Burkina Faso (approved 07/12/2016, ref: 2016-12-142) and from the International Food Policy Research Institute Institutional Review Board in Washington, DC (approved 26/12/2016, ref: IRB00007490). Informed consent was documented in writing from each household head prior to the interviews.

## Results

### Baseline characteristics and loss to follow-up

The dry season enrollment survey (round 1) included a total of 1054 households with index child and caregiver dyads in 45 communes across the 3 regions (Figure 1). Generally, there were no substantive differences between study groups at enrollment, except for income-generating activities conducted by women (**Table 1**).

**TABLE 1:** Characteristics of the study population at enrollment in SELEVER and control communities, Burkina Faso^1^

Characteristic	Control	Treatment	SELEVER	SELEVER + WASH
Target children	*n* = 356	*n* = 698	*n* = 353	*n* = 345
Age, mo	40 ± 9.7	41 ± 10	41 ± 10	41 ± 10
Male	50	50	48	52
Sick during the recall day	7.3	9.7	11	8.4
Women	*n* = 356	*n* = 698	*n* = 353	*n* = 345
Biological mother of child	99	98	98	98
Age, y	31 ± 7.0	31 ± 7.1	31 ± 7.6	31 ± 6.5
Married	96	96	96	96
Never been to formal school	82	82	79	85
Income-generating activity	34	26	23	29
Sick during the recall day	1.4	3.2	4.5	1.7
Breastfeeding	42	39	36	42
Pregnant	15	13	13	13
Has a child aged 0–24 mo	39	37	35	38
Has a child aged 6–24 mo	28	26	25	28
Households	*n* = 356	*n* = 698	*n* = 353	*n* = 345
HH age, y	44 ± 13	44 ± 14	43 ± 13	45 ± 14
HH is male	97	97	98	97
HH has never been to formal school	71	70	67	73
HH has income-generating activity	46	45	45	44
Household: moderate or severe hunger	4.8	3.7	2.4	4.9
Yesterday was a market day in the village	18	22	18	26

1 Descriptive values are unadjusted percentages or means ± SDs. HH, household head; SELEVER, Soutenir l'Exploitation Familiale pour Lancer l’Élevage des Volailles et Valoriser l’Économie Rurale; WASH, water, hygiene, and sanitation.

The lean season preintervention baseline survey (round 2) successfully tracked 98% of households (Figure 1). The overall attrition rate at round 3 was inferior to 15% for any study group. Attrition was significantly higher in the SELEVER + WASH group relative to the SELEVER group. However, there were no statistically significant differences between study groups at enrollment in the subsample of nonattrited children, except that the proportion of female primary caregivers with income-generating activity was significantly lower in the standard SELEVER group compared with the control group (**Supplemental Table 1**). We found the same baseline difference in the subsample of households with a IYC during the lean season endline survey (**Supplemental Table 2**).

For diet analyses, we excluded 2 caregivers at round 2 who both reported extreme portion sizes across several foods consumed, resulting in more than 22,000 kcal consumed over 24 h; 1 caregiver at round 2 who reported drinking only black coffee over 24 h (0 kcal) with no explanation; and 1 index child at round 2 who was reported sick and consumed only 67 kcal of milk over 24 h.

### Program exposure

In the 12 mo preceding the endline survey, 27% of households in treatment villages attended at least 1 training of each of the 3 themes (poultry, nutrition/gender, and WASH) through the participation of the father and/or mother of an index child, and hence were exposed to all 3 components of the intervention, compared with 2.3% of households in control villages (*P* < 0.001, **Table 2**).

**TABLE 2:** Exposure in the 12 mo prior to the lean season endline survey (round 3) of primary female and male caregivers of the index child to various services that may be provided within SELEVER, by study group^1^

	Round 3				Round 3				SELEVER + WASH		SELEVER + WASH	
	Control	Treatment	Treatment vs. control		SELEVER	SELEVER + WASH	SELEVER vs. control		vs. control		vs. SELEVER	
Characteristic	(*n* = 350)	(*n* = 661)	Δ pp	*P* value	(*n* = 333)	(*n* = 328)	Δ pp	*P* value	Δ pp	*P* value	Δ pp	*P* value
Father or mother heard of SELEVER	27	53	27	<0.001^* *^	50	56	26	<0.001^* *^	29	<0.001^* *^	3.1	0.69
Father or mother said HH benefited from SELEVER	2.0	27	22	<0.001^* *^	23	31	20	<0.001^* *^	25	<0.001^* *^	5.0	0.41
Poultry component												
Father or mother is in poultry-related group (raising + selling)	4.0	24	18	<0.001^* *^	20	27	14	<0.001^* *^	23	<0.001^* *^	9.3	0.10
Father or mother participated in a talk/group training on poultry raising	12	49	32	<0.001^* *^	45	52	30	<0.001^* *^	34	<0.001^* *^	4.6	0.46
Father and mother, number of poultry training talks attended	0.18	1.7	1.3	<0.001^* *^	1.6	1.7	1.3	<0.001^* *^	1.3	<0.001^* *^	−0.025	0.94
Father or mother benefited from any service at home provided by a VVV	59	66	8.0	0.25	62	69	5.9	0.47	10	0.20	4.3	0.58
Nutrition and gender component												
Father or mother is in nutrition group	5.8	15	8.7	<0.001^* *^	11	19	5.9	0.019*	12	0.0011^* *^	5.9	0.12
Father or mother is in gender group	2.9	14	11	<0.001^* *^	13	16	10	<0.001^* *^	13	<0.001^* *^	3.0	0.43
Father or mother attended meetings on nutrition or gender	19	39	19	<0.001^* *^	36	42	20	0.0010^* *^	19	<0.001^* *^	−1.1	0.86
Father and mother, number of nutrition/gender sessions	0.52	1.8	1.1	<0.001^* *^	1.5	2.0	1.1	<0.001^* *^	1.2	<0.001^* *^	0.15	0.73
Father or mother received a home visit to discuss about nutrition	4.9	14	7.0	0.0032^* *^	12	16	5.2	0.045*	8.8	0.0044^* *^	3.6	0.27
Father or mother received a home visit to discuss about women's role	3.2	12	6.9	0.0024^* *^	11	14	4.8	0.062	9.0	0.0021^* *^	4.2	0.21
WASH component												
Father or mother is in WASH group	10	27	17	<0.001^* *^	19	36	9.1	0.035*	26	<0.001^* *^	17	<0.001^* *^
Father or mother participated in group talks on WASH	28	51	25	<0.001^* *^	44	58	18	0.0035^* *^	32	<0.001^* *^	14	0.027*
Father or mother, number of WASH trainings attended	0.64	1.3	0.68	<0.001^* *^	1.1	1.5	0.54	0.012^* *^	0.81	<0.001^* *^	0.27	0.26
Father or mother received a home visit to discuss link between children'shealth and hygiene	11	24	12	0.0012^* *^	19	28	6.9	0.056	17	<0.001^* *^	10	0.056
Combination of multiple components												
Father or mother attended trainings on poultry and group talks onnutrition, and gender	3.2	31	25	<0.001^* *^	29	34	25	<0.001^* *^	25	<0.001^* *^	0.047	0.99
Father and mother, number of non-WASH sessions	0.70	3.4	2.5	<0.001^* *^	3.1	3.7	2.4	<0.001^* *^	2.5	<0.001^* *^	0.12	0.86
Father or mother attended trainings on poultry and group talks onnutrition, gender, and trainings on WASH	2.3	27	23	<0.001^* *^	25	29	23	<0.001^* *^	23	<0.001^* *^	−0.14	0.98
Father and mother, total number of sessions	1.3	4.7	3.1	<0.001^* *^	4.3	5.2	2.9	<0.001^* *^	3.3	<0.001^* *^	0.40	0.65

1 Descriptive values are unadjusted percentages. Δ is the linear regression coefficient and represents the change in the outcome in a group compared with another group, as indicated in column headers. Asterisks indicate *P* values of comparison of Δ to 0 value: *P < 0.05, which is the level of significance set for the study; ^* *^P < 0.017, which is the level of significance when adjusting for multiple testing across 3 study groups using the Bonferroni method. HH, ; pp, percentage points; SELEVER, Soutenir l'Exploitation Familiale pour Lancer l’Élevage des Volailles et Valoriser l’Économie Rurale; VVV, village volunteer vaccinator and popularizer; WASH, water, hygiene, and sanitation.

### Impact of the intervention (SELEVER and SELEVER + WASH) compared with control

Overall nutrient adequacy was low in all study groups and at all time points in both caregivers and index children (**Table 3**). The intervention increased the PA of iron intakes in women by 1.8 pp. The intervention had no impact on the PA of iron intakes in index children or on the PAs of vitamin A and zinc intakes, as well as the MPA in women and index children.

**TABLE 3:** Impact of SELEVER on the probability of adequate intakes of vitamin A, iron, and zinc, on the mean probability of adequacy and on IDDS, in caregivers and index children^1^

	Round 2		Round 3		Treatment vs. control		Round 2		Round 3		SELEVER vs. control		SELEVER + WASH vs. control		SELEVER + WASH vs. SELEVER	
Characteristic	Control	Treatment	Control	Treatment	Δ	*P* value	SELEVER	SELEVER + WASH	SELEVER	SELEVER + WASH	Δ	*P* value	Δ	*P* value	Δ	*P* value
Caregivers	*n* = 339	*n* = 673	*n* = 326	*n* = 630			*n* = 343	*n* = 330	*n* = 328	*n* = 302						
Energy intake, kcal/d	2007 ± 929	2151 ± 886	2127 ± 1163	2144 ± 984	63.7	0.56	2103 ± 878	2201 ± 893	2109 ± 885	2182 ± 1081	3.9	0.97	126	0.33	122	0.20
PA vitamin A, %	0.47 ± 5.1	0.57 ± 4.7	0.53 ± 5.8	0.49 ± 3.4	0.25	0.23	0.29 ± 2.6	0.85 ± 6.2	0.58 ± 4.3	0.39 ± 2.0	0.33	0.32	0.17	0.35	−0.16	0.64
PA iron, %	44 ± 10	43 ± 10	38 ± 10	37 ± 11	1.8	0.030*	43 ± 11	43 ± 10	36 ± 12	38 ± 10	1.7	0.059	1.9	0.035*	0.2	0.78
PA zinc, %	72 ± 40	64 ± 41	69 ± 42	64 ± 42	−2.0	0.25	60 ± 42	68 ± 40	60 ± 43	69 ± 41	−4.0	0.052	0.09	0.96	4.1	0.038*
MPA, %	22± 5.6	22 ± 5.9	20 ± 7.0	20± 6.9	0.37	0.48	21± 6.0	22± 5.8	19 ± 7.1	20 ± 6.5	0.034	0.95	0.70	0.21	0.67	0.17
IDDS,^2^ food groups	3.6 ± 0.94	3.6 ± 0.99	3.7 ± 0.96	3.8 ± 1.0	−0.060	0.59	3.6 ± 1.0	3.6 ± 0.99	3.8 ± 1.0	3.7 ± 1.0	−0.020	0.87	−0.10	0.49	−0.081	0.60
Index children	*n* = 342	*n* = 676	*n* = 321	*n* = 623			*n* = 345	*n* = 331	*n* = 326	*n* = 297						
Energy intake, kcal/d	1292 ± 628	1368 ± 574	1512 ± 669	1494 ± 642	5.4	0.9	1359 ± 562	1378 ± 588	1455 ± 627	1536 ± 656	−31	0.60	46	0.53	77	0.23
PA vitamin A, %	11 ± 23	12 ± 25	6.5 ± 19	7.1 ± 18	0.82	0.58	11 ± 23	13 ± 26	7.1 ± 19	7.2 ± 18	0.64	0.69	1.0	0.54	0.37	0.79
PA iron, %	90 ± 15	90 ± 15	84 ± 23	83 ± 24	−1.4	0.5	91 ± 14	90 ± 16	83 ± 24	82 ± 24	0.38	0.86	−3.3	0.21	−3.7	0.17
PA zinc, %	100 ± 0.90	100 ± 1.6	94 ± 16	93 ± 16	−1.4	0.38	100 ± 2.2	100 ± 0.70	94 ± 17	93 ± 16	−0.17	0.90	−2.7	0.27	−2.5	0.29
MPA, %	53 ± 10	53 ± 11	45 ± 14	45 ± 14	−0.34	0.77	53 ± 11	53 ± 11	45 ± 14	45 ± 15	0.30	0.81	−1.0	0.48	−1.3	0.36
IDDS,^2^ food groups	3.6 ± 0.96	3.6 ± 1.0	3.8 ± 0.95	3.8 ± 1.0	−0.048	0.63	3.6 ± 1.0	3.7 ± 1.0	3.9 ± 1.0	3.8 ± 0.99	−0.036	0.76	−0.062	0.61	−0.025	0.85

1 Descriptive values are unadjusted means with standard deviation. Δ is the linear regression coefficient and represents the change in the outcome in a group compared with another group, as indicated in column headers. It is expressed in the same unit as the outcome or, when the unit is a percentage, it is expressed in percentage points. Asterisks indicate *P* values of comparison of Δ to 0 value: *P < 0.05, which is the level of significance set for the study; ^* *^P < 0.017, which is the level of significance when adjusting for multiple testing across 3 study groups using the Bonferroni method. IDDS, Individual Dietary Diversity Score; MPA, mean probability of adequacy; PA, probability of adequacy; SELEVER, Soutenir l'Exploitation Familiale pour Lancer l’Élevage des Volailles et Valoriser l’Économie Rurale; VVV, village volunteer vaccinator and popularizer; WASH, water, hygiene, and sanitation.

2 Number of food groups consumed the previous day of 10 food groups: starchy staples; pulses; nuts and seeds; dairy; meat, poultry, and fish; eggs; dark green leafy vegetables; other vitamin A–rich fruit and vegetables; other vegetables; and other fruit.

In IYC, IYCF indicators were also suggestive of poor diets at baseline, with <15% of the sample meeting minimum acceptable diet; the intervention had no impact on this primary indicator (**Table 4**).

**TABLE 4:** Impact of SELEVER on minimum acceptable diet and other infants and young children (IYC) feeding indicators in children 6–23 mo of age of the IYC repeated cross-sectional samples^1^

	Round 2		Round 3				Round 2		Round 3		SELEVER		SELEVER + WASH		SELEVER + WASH	
	Control	Treatment	Control	Treatment	Treatment vs. control		SELEVER	SELEVER + WASH	SELEVER	SELEVER + WASH	vs. control		vs. control		vs. SELEVER	
Characteristic	(*n* = 108)	(*n* = 197)	(*n* = 99)	(*n* = 186)	Δ	*P* value	(*n* = 89)	(*n* = 108)	(*n* = 99)	(*n* = 87)	Δ	*P* value	Δ	*P* value	Δ	*P* value
Minimum acceptable diet	9.3	15	14	19	2.8	0.47	16	15	17	22	0.38	0.93	5.9	0.22	5.5	0.27
IDDS,^2^ food groups	2.5 ± 1.1	2.7 ± 1.2	2.6 ± 1.2	2.8 ± 1.2	0.25	0.17	2.9 ± 1.2	2.6 ± 1.2	2.6 ± 1.1	3.1 ± 1.3	0.054	0.77	0.46	0.025*	0.41	0.030*
Minimum dietary diversity	15	19	20	24	1.0	0.86	23	17	19	30	−4.1	0.48	6.5	0.31	11	0.11
Minimum meal frequency	49	57	47	55	11	0.21	53	59	59	51	19	0.046*	1.1	0.91	−18	0.028*
Consumption of iron-rich foods/supplements	23	24	26	31	0.67	0.94	24	24	26	37	−7.3	0.39	9.2	0.33	17	0.051

1 Descriptive values are unadjusted percentages or unadjusted means with standard deviation. Δ is the linear regression coefficient and represents the change in the outcome in a group compared with another group, as indicated in column headers. It is expressed in the same unit as the outcome or, when the unit is a percentage, it is expressed in percentage points. Asterisks indicate *P* values of comparison of Δ to 0 value: *P  < 0.05, which is the level of significance set for the study; ^* *^P < 0.017, which is the level of significance when adjusting for multiple testing across 3 study groups using the Bonferroni method. IDDS, Individual Dietary Diversity Score; SELEVER, Soutenir l'Exploitation Familiale pour Lancer l’Élevage des Volailles et Valoriser l’Économie Rurale; WASH, water, hygiene, and sanitation.

2 Number of food groups consumed the previous day of 7 food groups: starchy staples, legumes and nuts, dairy, flesh foods, eggs, vitamin A–rich fruit and vegetables, other fruit and vegetables.

We also looked at the impact of the SELEVER interventions on secondary dietary indicators, including the prevalence of consumption of food groups as promoted by the program BCC (**Table 5**), the prevalence of consumption and quantities consumed of food groups according to standard classifications (**Supplemental Tables 3** and **4**), other IYCF practices besides minimum acceptable diet (Table 4), and the quantities of nutrients intakes and PAs of the 8 other micronutrients used to calculate MPA (**Supplemental Table 5**). We found a significant increase in egg consumption in index children (in terms of both prevalence and quantity consumed) and a significant increase of the prevalence of IYC who consumed all 3 promoted food groups in the previous 24 h but no further impact of the SELEVER interventions.

**TABLE 5:** Impact of SELEVER on consumption of the 3 food groups used in the behavior change communication strategy in caregivers, index children and IYC^1^

	Round 2		Round 3		Treatment vs. control		Round 2		Round 3		SELEVER vs. control		SELEVER + WASH vs. control		SELEVER + WASH vs. SELEVER	
Characteristic	Control	Treatment	Control	Treatment	Δ pp	*P* value	SELEVER	SELEVER + WASH	SELEVER	SELEVER + WASH	Δ pp	*P* value	Δ pp	*P* value	Δ pp	*P* value
Caregivers	*n* = 339	*n* = 673	*n* = 320	*n* = 622			*n* = 343	*n* = 330	*n* = 325	*n* = 297						
Energy giving	100	100	100	100	−0.098	0.33	100	100	100	100	−0.011	0.50	−0.19	0.33	−0.18	0.33
Protective	96	95	98	98	−1.2	0.12	96	95	99	97	−0.37	0.65	−2.1	0.080	−1.8	0.19
Body building	83	84	85	84	−6.1	0.15	83	86	85	82	−4.3	0.32	−8.0	0.16	−3.7	0.50
All food groups	80	80	84	82	−6.3	0.14	79	82	85	80	−3.6	0.41	−9.2	0.10	−5.6	0.31
Index children	*n* = 342	*n* = 676	*n* = 316	*n* = 621			*n* = 345	*n* = 331	*n* = 326	*n* = 295						
Energy giving	100	100	100	100	−0.032	0.33	100	100	100	100	−0.0060	0.36	−0.061	0.32	−0.055	0.32
Protective	97	95	99	98	−1.3	0.085	96	95	99	98	−0.72	0.33	−1.9	0.13	−1.2	0.39
Body building	85	85	87	84	−6.1	0.091	84	86	84	85	−6.4	0.11	−5.8	0.23	0.65	0.90
All food groups	83	81	86	83	−6.9	0.059	80	82	83	83	−6.4	0.11	−7.4	0.12	−1.1	0.83
IYC	*n* = 108	*n* = 197	*n* = 99	*n* = 186			*n* = 89	*n* = 108	*n* = 99	*n* = 87						
Energy giving	98	98	98	98	−0.92	0.63	100	97	96	100	−3.2	0.29	1.6	0.14	4.8	0.089
Protective	70	75	76	76	3.8	0.57	83	69	75	78	3.8	0.65	3.8	0.59	−0.044	1.0
Body building	53	52	54	62	12	0.17	51	52	55	70	6.4	0.48	19	0.08	12	0.18
All food groups	37	36	32	46	12	0.040*	38	34	42	49	12	0.091	12	0.092	0.44	0.96

1 Descriptive values are unadjusted percentages. Δ is the linear regression coefficient and represents the change in the outcome in a group compared with another group, as indicated in column headers. Asterisks indicate *P* values of comparison of Δ to 0 value: *P < 0.05, which is the level of significance set for the study; ^* *^P < 0.017, which is the level of significance when adjusting for multiple testing across 3 study groups using the Bonferroni method. IYC, infants and young children; pp, percentage points; SELEVER, Soutenir l'Exploitation Familiale pour Lancer l’Élevage des Volailles et Valoriser l’Économie Rurale; WASH, water, hygiene, and sanitation.

### Second-level comparison of the 3 study groups (SELEVER compared with SELEVER + WASH compared with control)

When examining comparisons of both primary and secondary indicators across the 3 treatment groups, we found either no differences across groups or some negative results in the SELEVER group compared with either the control group (quantity of nuts and seeds consumed and quantity of zinc intakes in children and IDDS in IYC) or the SELEVER + WASH group (prevalence of consumption of pulses, quantity of protein consumed, and PA of zinc intakes in women; quantity of iron consumed in children; and prevalence of flesh foods intake and IDDS in IYC) (Tables 3–5, Supplemental tables 3–5). On the other hand, in IYC, the prevalence of consumption of oils and fats and the minimum meal frequency were greater in the SELEVER group compared with either the control or the SELEVER + WASH group. There were no differences between the SELEVER + WASH group and the control group except for a positive effect of SELEVER + WASH on the PA of iron intakes in women and on IDDS in IYC. Most of these differences were not significant at the revised level of significance of 1.7%.

## Discussion

To our knowledge, this study is among the first cluster randomized control trials evaluating the effectiveness on diets of using a poultry value chain platform to improve diets of women and children during the lean season in rural, food-insecure settings and the first study that has measured the impact on diet adequacy. The rigorous evidence presented in this article suggests that during the lean season, the integrated agriculture and nutrition interventions, incorporating training to improve poultry production systems and market facilitation, alongside BCC on improved diets and women's empowerment, had negligible to no effect on the adequacy of micronutrient intakes for women and children aged 2–4 y or on appropriate complementary feeding.

Participation to the intervention was offered to anyone interested in the community, and the intervention relied mainly on community engagement and self-selection for trainings. The moderate program exposure in our sample may partially explain its limited impact. Both coverage and quality of counseling have been shown to be crucial to nutrition impact of BCC interventions (36). The moderate coverage may be reflective of an insufficient incentive power of this type of intervention for alleviating other barriers to program participation. In addition, previous evidence is suggestive that economic constraints, and not information constraints, may be binding during the lean season, and thus information alone may be necessary but not sufficient to behavior change (10). Further evidence from implementation research on poultry‐for‐nutrition projects, including asset transfers in 4 African countries, recognized that practices are challenging to increase, including egg consumption, as well as best poultry‐rearing practices and productivity (37). This seemed to be the case for SELEVER, as households exposed to the intervention significantly increased their use of poultry inputs and reported higher revenue; however, there was no evidence of an increase in profits in the lean season (16). Therefore, we expected the impact of the intervention on diet during the lean season to be lower bounds of the potential effectiveness of the BCC intervention during the postharvest season, when household economic resources are less constrained.

Our results are in line with this hypothesis, as we found very few dietary improvements overall. At the population level, the average PA of a given micronutrient is equivalent to a prevalence of adequacy of intake of this micronutrient. We found that the prevalence of adequate iron intakes in women improved by <2 pp, which does not seem meaningful at the population level. Furthermore, this was not supported by significant improvements in iron intakes or in intakes of iron-rich food groups. Likewise, the magnitude of the improvement in egg consumption in children (<1 pp and 1 kcal/d) was far below what is required to improve diet adequacy (38). Results from the impact evaluation of a nutrition-sensitive poultry production intervention in Ethiopia further support, in the specific context of a poultry value chain, the hypothesis that nutrition BCC is necessary, but not sufficient, to affect diets in the lean season (11). Indeed, a positive impact on overall child diet diversity was observed only in the arm integrating nutrition BCC in addition to the poultry production intervention; however, this was observed only in the season of moderate food security (endline) but not in the season of lowest food security (midline). It must be acknowledged that the Ethiopia intervention involved a transfer of 25 chicks per household, which may have partially alleviated some of the economic constraints on beneficiaries. Nevertheless, in our setting, it remains unknown if the negligible impacts on diet indicators in the lean season would increase in the dry season. This hypothesis will be examined explicitly on completion of the randomized trial.

Beyond the overall limited impact of the SELEVER intervention, our analysis highlighted unexpected differences across the 2 SELEVER implementation groups. First, some significant results are suggestive that the standard SELEVER intervention implemented without the additional WASH component had some negative effects on diets during the lean season relative to the control group or to the SELEVER + WASH group. Although these generally small to moderate differences did not result in significant differences in nutrient intakes, the fact they were negative warrants attention. One hypothesis is that they may result from the nutrition BCC strategy focusing on the promotion of 3 food groups (energy-giving, body-building, and protective foods) to diversify diets. Baseline data showed that most caregivers and index children were already consuming every day all 3 food groups promoted through BCC, with the building food group being the only group with little room for improvement (Table 5). The intervention may have inadvertently sensitized communities with the messaging that daily consumption of all these 3 food groups was adequate in terms of having a healthy diet, leading to intervention households not diversifying *within* the 3 food groups and resulting in decreased overall diet diversification. The lack of within-group diversification is most relevant within the body-building foods. This is also where we observed negative effects, although negatively affected food groups varied across women (pulses), children (nuts and seeds), and infants (flesh foods). These particular findings have important policy and program design implications for BCC interventions in these low-income contexts. For example, in Chad, the officially validated flipchart widely used to deliver IYCF BCC is based on this 3 food groups classification (39).

Nevertheless, as both SELEVER groups received the BCC intervention, this first hypothesis cannot explain alone why the SELEVER + WASH intervention was effective relative to the standard SELEVER intervention with regards to *not decreasing* diet outcomes. Then, 3 main program-related explanations for this result are possible, including *1*) intervention design features providing additional nutrition information in the WASH intervention, *2*) differential exposure to nutrition BCC because of additional WASH activities (synergized implementers), and *3*) synergies between nutrition BCC and WASH activities (synergized beneficiaries). From the intervention design perspective, the additional poultry-WASH–related activities did not include additional diet-related information. Rather, the activities focused on providing more intensive exposure to hygiene-related messaging, including community-level sensitization on the potential harmful effects on health of open defecation and livestock feces. The second hypothesis on the synergies between implementers, who, because of the additional community-level WASH activities, were able to coordinate more effectively at community level, was suggested during the process evaluation (40). In this analysis, we had only suggestive evidence that additional WASH activities might have increased coverage or intensity of exposure to other SELEVER interventions, as the coefficients for program exposure indicators reported were consistently, but not significantly, slightly favoring SELEVER + WASH compared with standard SELEVER. Also, the additional WASH activities may have somewhat attenuated the negative effects of SELEVER by diluting some of the messaging on the 3 food groups or by highlighting the importance of improving child nutrition more broadly, including diets, hygiene, and health.

The main strength of this study relies on the rigorous experimental design. One important limitation is that the indicators reported in this analysis rely on self-reported dietary assessment and may thus suffer from respondent and enumerator bias (41). We intended to limit this constraint through prior notice and explanation to the respondents and the provision of standard plates and bowls, intense training and supervision of enumerators, and the use of a user-friendly computer-assisted personal interview giving the necessary probes and indications for the enumerator and the respondent to finely describe food items and use appropriate method(s) to quantify portions. Furthermore, there is no reason to believe that if bias occurred, it was different across study groups. An impact analysis of objective anthropometric and/or biochemical indicators of the nutritional status in our 3 samples is under way. However, such objective nutrition indicators are determined by a larger set of factors than just food consumption, and previous evidence in our study context confirms that they are not relevant to approximate diets (21).

Our findings have some important policy implications. First, the rigorous evidence presented in this study suggests that information interventions that aim to improve diets in rural food-insecure settings may not have a sufficient incentive power to largely expose communities and may not be effective at the population level without additional transfers to alleviate economic constraints, especially in the lean season. Second, synergies between inappropriate diet diversification BCC and the WASH intervention might have affected the intensity or salience of the BCC messaging or of other SELEVER interventions. This highlights the potential to improve interventions’ effectiveness through better design of the BCC strategy and better understanding of the synergies across multiple intervention components.

## Supplementary Material

nxac034_Supplemental_File

## Notes

Supported by the Bill & Melinda Gates Foundation (grant OPP1149709). We also acknowledge support from the CGIAR Research Program on Agriculture for Nutrition and Health (A4NH), led by the International Food Policy Research Institute (IFPRI; subgrant A4NH-202004.040.500). Freedom to publish the study findings was protected contractually in the agreement between the respective funding sources and IFPRI. All final decisions on the manuscript were made by the researchers.

Author disclosures: The authors report no conflicts of interest.

Supplemental Tables 1–5 are available from the “Supplementary data” link in the online posting of the article and from the same link in the online table of contents at https://academic.oup.com/jn/.

Abbreviations used: BCC, behavior change communication; FCT, food composition table; IDDS, Individual Dietary Diversity Score; IYC, infants and young children; IYCF, infant and young child feeding; MPA, mean probability of adequacy; NGO, nongovernmental organization; PA, probability of adequacy; pp, percentage points; SELEVER, Soutenir l'Exploitation Familiale pour Lancer l’Élevage des Volailles et Valoriser l’Économie Rurale; SELEVER + WASH, SELEVER interventions with a hygiene and sanitation intervention; WASH, water, hygiene, and sanitation.
