# Supplementary material for: A Poultry Value Chain Intervention Promoting Diversified Diets Has Limited Impact on Maternal and Child Diet Adequacy during the Lean Season in a Cluster Randomized Controlled Trial
Source: J Nutr. 2022 Feb 16;152(5):1336–46. doi: 10.1093/jn/nxac034 (PMC9071289; doi:10.1093/jn/nxac034)
Supplement: nxac034_Supplemental_File [file nxac034_supplemental_file.zip › JN_Diet SELEVER lean season_2022-03-17_Online material_clean.rtf]

Becquey et al. A poultry value chain intervention promoting diversified diets had limited impact on maternal and child diet adequacy during the lean season in a cluster randomized controlled trial. Online Supplementary Material
Supplementary table 1. Comparison across study groups of characteristics at enrollment (round 1) in households of non-attrited children during the lean season endline (round 3)1

		Control	Treatment	SELEVER	SELEVER+WASH	
Children		n=316	n=621	n=326	n=295	
Age, mo	40 ± 9.6	41 ± 10	41 ± 10	41 ± 10 	
	Male	52%	52%	48%	56%	
Sick during the recall day	7.3%	10%	10%	9.5%	
Women		n=316	n=621	n=326	n=295	
Biological mother of child	99%	98%	98%	98%	
	Age, y	31 ± 7.0	31 ± 7.0	31 ± 7.5	31 ± 6.4 	
	Married	97%	96%	96%	97%	
Never been to formal school	82%	83%	80%	86%	
Income generating activity	33%	25%	23%	29%	
Sick during the recall day	1.6%	3.4%	4.6%	2.0%	
Breastfeeding 	42%	39%	36%	42%	
	Pregnancy	17%	14%	13%	14%	
Has a child aged 0-24 months	39%	36%	35%	38%	
Has a child aged 6-24 months	29%	26%	25%	28%	
Households/ Household head, HH)	n=316	n=621	n=326	n=295	
	HH age, y	44 ± 12	45 ± 13	44 ± 13 	46 ± 14	
	 HH is male	98%	98%	98%	98%	
	HH has never been to formal school	71%	70%	68%	73%	
	HH has income generating activity	46%	45%	45%	44%	
Moderate or severe hunger	5.1%	3.1%	2.1%	4.1%	
Yesterday was a market day in the village	19%	21%	17%	27%	
1Descriptive values are unadjusted percentages or means with standard deviations. 


Supplementary table 2. Comparison across study groups of characteristics at enrollment (round 1) in households with an eligible IYC during the lean season endline (round 3)1
		Control	Treatment	SELEVER	SELEVER+WASH	
Children		n=183	n=351	n=169	n=182	
Age, mo	42 ± 9.2 	44 ± 9.2	44 ± 9.1	43 ± 9.2 	
	Male	47%	47%	43%	51%	
Sick during the recall day	8.7%	9.7%	9.5%	9.9%	
Women		n=183	n=351	n=169	n=182	
Biological mother of child	100%	98%	99%	98%	
	Age, y	30 ± 6.3 	30 ± 6.7	30 ± 6.7	31 ±6.7	
	Married	98%	97%	97%	98%	
Never been to formal school	81%	84%	82%	86%	
Income generating activity	30%	25%	21%	29%	
Breastfeeding 	72%	66%	65%	67%	
	Pregnancy	6.0%	8.0%	5.9%	9.9%	
Has a child aged 0-24 months	75%	72%	72%	73%	
Has a child aged 6-24 months	54%	52%	51%	52%	
Households/ Household head, HH)	n=183	n=351	n=169	n=182	
	HH age, y	43 ± 12	44 ± 13	43 ± 12 	45 ± 14	
	 HH is male	97%	99%	100%	99%	
	HH has never been to formal school	74%	72%	70%	74%	
	HH has income generating activity	48%	44%	46%	43%	
Moderate or severe hunger	3.3%	3.4%	2.4%	4.4%	
Yesterday was a market day in the village	15%	23%	19%	26%	
1Descriptive values are unadjusted percentages or means with standard deviations. 


Supplementary table 3. Impact of SELEVER on food group consumption in caregivers, index children, and their younger 6-23 months old siblings 1

	Round 2	Round 3	Treatment versus Control	Round 2	Round 3	SELEVER 
versus Control	SELEVER+WASH versus Control	SELEVER+WASH
versus SELEVER		
	Control	Treatment	Control	Treatment	Ä pp	P-value	SELEVER	SELEVER
+Wash	SELEVER	SELEVER
+Wash	Ä pp	P-value	Ä pp	P-value	Ä pp	P-value		
Caregivers	n=339	n=673	n=320	n=622	 	 	n=343	n=330	n=325	n=297	 	 	 	 	 	 		
Starchy staples	100	100	100	100	-0.10	0.33	99	100	100	100	-0.010	0.51	-0.19	0.33	-0.18	0.33		
Pulses	30	28	35	31	-4.4	0.40	26	30	25	38	-9.9	0.085	1.2	0.82	11	0.023*		
Nuts and seeds	71	72	76	75	-3.2	0.53	73	70	80	70	2.0	0.71	-8.5	0.17	-11	0.10		
Dairy	8.6	6.8	8.1	7.9	0.86	0.65	7.6	6.1	7.7	8.1	0.26	0.89	1.5	0.57	1.2	0.63		
Meat, poultry and fish	17	18	14	15	-1.7	0.70	18	18	16	14	-0.91	0.86	-2.5	0.63	-1.6	0.76		
Eggs	0.30	0.30	0.00	0.81	0.54	0.067	0.29	0.30	0.31	1.4	0.51	0.24	0.56	0.12	0.049	0.93		
Dark green leafy vegetables	58	60	60	66	2.8	0.63	61	58	69	62	6.8	0.23	-1.2	0.87	-8.0	0.21		
Other vitamin A-rich fruits and vegetables	0.59	3.6	0.63	0.97	0.39	0.29	1.2	6.1	1.5	0.34	0.57	0.26	0.19	0.67	-0.39	0.53		
Other vegetables	73	71	78	80	-0.89	0.87	70	72	80	79	-0.60	0.91	-1.2	0.86	-0.59	0.91		
Other fruits	0.00	1.0	0.63	0.81	0.74	0.35	1.7	0.30	0.62	1.0	0.0090	0.99	1.5	0.25	1.5	0.31		
Minimum dietary diversity	15	16	22	22	-3.8	0.31	16	16	22	22	-3.7	0.38	-3.8	0.38	-0.11	0.98		
Index children	n=342	n=676	n=316	n=621		 	n=345	n=331	n=326	n=295								
Starchy staples	100	100	100	100	-0.032	0.33	99	100	100	100	-0.0060	0.36	-0.061	0.32	-0.055	0.32		
Pulses	29	29	35	34	-3.1	0.55	26	32	28	40	-8.4	0.14	2.5	0.68	11	0.058		
Nuts and seeds	74	72	77	76	-3.5	0.46	72	72	80	72	0.44	0.93	-7.7	0.19	-8.1	0.16		
Dairy	10	8.3	8.2	8.5	0.86	0.67	10	6.6	8.6	8.5	0.34	0.87	1.4	0.59	1.1	0.66		
Meat, poultry and fish	17	18	14	15	-1.3	0.79	19	18	15	14	-1.0	0.85	-1.7	0.77	-0.67	0.89		
Eggs	0.29	0.59	0.00	1.3	0.73	0.010**	0.58	0.60	0.61	2.0	0.68	0.11	0.79	0.063	0.11	0.86		
Dark green leafy vegetables	60	60	61	66	2.9	0.62	62	58	69	63	5.5	0.33	0.0030	1.0	-5.5	0.32		
Other vitamin A-rich fruits and vegetables	0.88	3.7	0.95	1.3	0.42	0.43	1.4	6.0	1.5	1.0	0.45	0.40	0.37	0.65	-0.077	0.93		
Other vegetables	73	71	79	79	-2.0	0.69	70	72	81	78	-1.2	0.80	-2.8	0.67	-1.6	0.79		
Other fruits	0.88	2.4	2.5	2.4	1.4	0.32	3.8	0.91	1.5	3.4	0.0090	1.0	2.9	0.16	2.9	0.22		
Minimum dietary diversity	18	18	23	24	-3.5	0.40	18	18	25	23	-2.0	0.68	-5.1	0.24	-3.1	0.48		
IYC	n=108	n=197	n=99	n=186			n=89	n=108	n=99	n=87								
Starchy staples	98	98	94	94	1.9	0.57	99	97	93	95	1.2	0.79	2.8	0.41	1.6	0.66	
Legumes and nuts	26	28	26	34	8.3	0.27	29	28	30	39	3.5	0.65	14	0.18	10	0.32	
Dairy products	10	16	15	19	3.0	0.65	16	16	14	25	-0.38	0.95	7.0	0.41	7.4	0.34	
Flesh foods	24	27	30	32	-1.8	0.84	25	29	25	41	-12	0.20	10	0.37	22	0.031*	
Eggs	1.0	0.00	0.00	1.8	1.4	0.13	0.00	0.00	2.2	1.3	2.3	0.15	0.33	0.39	-1.9	0.23	
Vitamin A-rich fruits and vegetables	35	43	52	50	0.74	0.91	52	36	42	59	-3.3	0.64	5.1	0.51	8.4	0.25	
Other fruits and vegetables	56	58	44	49	3.8	0.61	65	52	51	46	5.6	0.54	1.7	0.83	-4.0	0.66	
Minimum dietary diversity 	15	19	20	24	1.0	0.86	23	17	19	30	-4.1	0.48	6.5	0.31	11	0.11	
Total oils and fats	27	34	32	35	7.7	0.35	36	32	40	30	18	0.035*	-4.0	0.67	-22	0.0060**	
1 Descriptive values are unadjusted percentages. Ä is the linear regression coefficient and represents the change in the outcome in a group versus another group, as indicated in column headers, expressed in percentage points.  Stars indicate p-values of comparison of Ä to 0 value: * means p-value <0.05, which is the level of significance set for the study; ** means p-value <0.017, which is the level of significance when adjusting for multiple testing across 3 study groups using the Bonferroni method. Abbreviations used: IYC, infants and young children


Supplementary table 4. Impact of SELEVER on quantities consumed, expressed in kcal, of 10 food groups in caregivers and index children1
 
	Round 2	Round 3	Treatment versus Control	Round 2	Round 3	SELEVER 
versus Control	SELEVER+WASH versus Control	SELEVER+WASH
versus SELEVER	
	Control	Treatment	Control	Treatment	Ä kcal	P-value	SELEVER	SELEVER
+Wash	SELEVER	SELEVER
+Wash	Ä kcal	P-value	Ä kcal	P-value	Ä kcal	P-value	
Caregivers	n=339	n=673	n=320	n=622	 	 	n=343	n=330	n=325	n=297							
Starchy staples	1297	1434	1352	1423	53	0.51	1,395	1,473	1,450	1,394	86	0.31	18	0.85	-68	0.40	
	(622)	(616)	(641)	(620)			(630)	(599)	(615)	(625)							
Pulses	170	154	162	162	7.0	0.81	144	164	120	208	-28	0.33	43	0.28	71	0.055	
	(344)	(317)	(279)	(319)			(311)	(323)	(252)	(373)							
Nuts and seeds	103	149	75	70	-7.6	0.74	157	140	59	82	-21	0.31	6.9	0.81	28	0.23	
	(226)	(319)	(177)	(168)			(363)	(266)	(138)	(195)							
Dairy	25	20	14	14	3.2	0.51	22	17	14	15	1.2	0.82	5.3	0.36	4.1	0.45	
	(116)	(95)	(78)	(78)			(99)	(92)	(85)	(70)							
Meat, poultry and fish	52	50	28	57	39	0.21	43	57	42	73	25	0.21	54	0.28	29	0.50	
	(218)	(195)	(117)	(398)			(133)	(242)	(169)	(547)							
Eggs	0.030	1.3	0.00	0.93	0.53	0.083	0.13	2.4	0.17	1.8	0.29	0.25	0.79	0.17	0.51	0.42	
	(0.6)	(31)	(0.0)	(14)			(2.4)	(44)	(3.0)	(18)							
Dark green leafy vegetables	34	39	38	44	4.6	0.55	37	42	44	44	6.3	0.42	2.7	0.76	-3.6	0.62	
	(58)	(71)	(63)	(63)			(59)	(82)	(64)	(63)							
Other vitamin A-rich fruits and vegetables	0.00	0.68	1.0	3.7	1.6	0.30	0.74	0.62	6.9	0.16	3.3	0.26	-0.19	0.62	-3.5	0.23	
	(0.0)	(12)	(18)	(52)			(14)	(9.2)	(72)	(2.7)							
Other vegetables	33	35	42	38	-3.6	0.57	33	37	36	41	-6.9	0.31	-0.15	0.99	6.7	0.37	
	(47)	(58)	(48)	(52)			(58)	(57)	(53)	(50)							
Other fruits	0.00	3.4	1.4	2.1	0.91	0.59	6.6	0.077	0.47	4.0	-1.3	0.24	3.2	0.35	4.5	0.23	
	(0.0)	(54)	(19)	(40)			(75)	(1.4)	(8.4)	(57)							
Index children	n=342	n=676	n=316	n=621			n=345	n=331	n=326	n=295							
Starchy staples	814	877	994	990	12	0.82	865	890	992	988	18	0.74	4.7	0.93	-13	0.75	
	(384)	(384)	(465)	(463)			(389)	(378)	(470)	(456)							
Pulses	106	100	106	109	9.8	0.62	93	107	89	131	-9.7	0.61	31	0.25	41	0.11	
	(246)	(208)	(180)	(191)			(199)	(217)	(176)	(205)							
Nuts and seeds	99	113	73	57	-25	0.17	117	108	48	66	-35	0.049*	-15	0.51	20	0.24	
	(246)	(208)	(180)	(191)			(199)	(217)	(176)	(205)							
Dairy	20	17	11	11	2.8	0.48	21	12	11	12	0.63	0.89	5.1	0.25	4.5	0.29	
	(83)	(65)	(66)	(58)			(75)	(53)	(59)	(56)							
Meat, poultry and fish	33	30	25	29	8.4	0.41	27	33	26	31	11	0.44	5.3	0.65	-6.0	0.72	
	(137)	(108)	(114)	(139)			(90)	(124)	(100)	(173)							
Eggs	0.040	0.93	0.00	1.2	0.69	0.036**	0.28	1.6	0.54	2.0	0.57	0.14	0.82	0.15	0.25	0.73	
	(0.7)	(19)	(0.0)	(14)			(3.6)	(27)	(7.4)	(19)							
Dark green leafy vegetables	27	30	31	37	3.3	0.60	30	30	37	36	4.8	0.45	1.6	0.83	-3.2	0.61	
	(45)	(51)	(51)	(59)			(42)	(59)	(60)	(59)							
Other vitamin A-rich fruits and vegetables	0.48	0.43	1.1	1.7	0.95	0.35	0.55	0.30	2.6	0.69	1.8	0.33	0.073	0.91	-1.7	0.37	
	(8.9)	(5.9)	(12)	(25)			(7.3)	(3.9)	(34)	(7.0)							
Other vegetables	26	27	34	32	-1.7	0.72	25	30	30	33	-3.0	0.60	-0.30	0.96	2.7	0.66	
	(38)	(41)	(40)	(43)			(36)	(46)	(44)	(41)							
Other fruits	0.21	4.2	2.9	3.9	2.8	0.26	6.4	1.8	1.0	7.0	-0.21	0.87	5.9	0.19	6.1	0.17	
	(3.8)	(38)	(29)	(34)			(48)	(24)	(9.7)	(47)							
1Descriptive values are unadjusted quantities consumed expressed in kcal, with standard deviation below the mean in parenthesis. Ä is the linear regression coefficient and represents the change in the outcome in a group versus another group, as indicated in column headers, expressed in kcal. Stars indicate p-values of comparison of Ä to 0 value: * means p-value <0.05, which is the level of significance set for the study; ** means p-value <0.017, which is the level of significance when adjusting for multiple testing across 3 study groups using the Bonferroni method. 


Supplementary table 5. Impact of SELEVER on intake of nutrients and probability of adequate micronutrients intakes in caregivers and index children1
	Round 2	Round 3 
 	Treatment-Control	Round 2	Round 3 	SELEVER 
versus Control	SELEVER+WASH versus Control	SELEVER+WASH
versus SELEVER	
	Control	Treatment	Control	Treatment	Ä	P-value	SELEVER	SELEVER
+Wash	SELEVER	SELEVER
+Wash	Ä	P-value	Ä	P-value	Ä	P-value	
Caregivers	n=339	n=673	n=326	n=630			n=343	n=330	n=328	n=302							
Vitamin A, ìg RAE	248	223	225	199	14	0.86	209	239	216	180	45	0.64	-17	0.82	-62	0.38	
	(584)	(883)	(610)	(493)			(583)	(1,113)	(528)	(452)							
Iron, mg	22	22	23	20	-1.7	0.48	21	24	18	21	-3.5	0.20	0.070	0.98	3.5	0.060	
	(17)	(18)	(25)	(16)			(16)	(19)	(14)	(17)							
Zinc, mg	9.2	10	10	9.1	-0.31	0.58	9.4	10	9.0	9.2	-0.52	0.35	-0.090	0.89	0.43	0.35	
	(4.9)	(4.6)	(5.9)	(4.5)			(4.4)	(4.7)	(4.3)	(4.8)							
Protein, g	62	64	62	61	1.3	0.69	61	67	58	65	-2.7	0.39	5.5	0.18	8.2	0.026*	
	(44)	(35)	(35)	(35)			(32)	(38)	(28)	(40)							
Thiamin, mg	0.85	0.88	0.90	0.79	-0.061	0.37	0.86	0.89	0.75	0.83	-0.11	0.11	-0.0090	0.91	0.10	0.11	
	(0.6)	(0.7)	(0.7)	(0.5)			(0.6)	(0.7)	(0.5)	(0.6)							
Riboflavin, mg	0.72	0.70	0.78	0.61	-0.072	0.35	0.66	0.74	0.58	0.65	-0.12	0.14	-0.026	0.76	0.092	0.14	
	(0.6)	(0.7)	(0.8)	(0.5)			(0.5)	(0.8)	(0.5)	(0.5)							
Niacin, mg	7.0	7.6	8.3	7.2	-0.35	0.60	7.5	7.7	7.2	7.1	-0.67	0.27	-0.012	0.99	0.66	0.42	
	(4.5)	(5.9)	(7.5)	(5.0)			(6.3)	(5.4)	(5.0)	(5.0)							
Vitamin B6, mg	0.99	1.0	1.0	0.93	-0.012	0.88	0.96	1.1	0.93	0.93	-0.047	0.57	0.023	0.79	0.071	0.26	
	(0.6)	(0.8)	(0.9)	(0.6)			(0.6)	(0.9)	(0.6)	(0.6)							
Folate, ìg	244	259	253	217	-13	0.59	250	268	204	230	-33	0.16	7.7	0.79	41	0.067	
	(218)	(249)	(260)	(193)			(239)	(258)	(171)	(213)							
Vitamin C, mg	22	24	26	29	8.8	0.23	22	25	24	34	2.4	0.72	15	0.25	13	0.39	
	(41)	(55)	(63)	(158)			(42)	(66)	(45)	(223)							
Calcium, mg	382	364	422	330	-36	0.55	355	374	319	342	-59	0.34	-12	0.85	47	0.27	
	(421)	(491)	(686)	(366)			(470)	(512)	(350)	(383)							
Vitamin B12, ìg	0.29	0.32	0.49	0.45	0.20	0.49	0.29	0.34	0.49	0.40	0.31	0.52	0.10	0.72	-0.21	0.68	
	(0.8)	(1.1)	(1.7)	(3.2)			(0.7)	(1.4)	(4.2)	(1.6)							
PA Thiamin, %	93	92	83	84	2.3	0.16	92	93	82	85	2.5	0.16	2.1	0.26	-0.39	0.81	
	(9.3)	(12)	(18)	(21)			(12)	(12)	(24)	(19)							
PA Riboflavin, %	0.010	0.010	0.020	0.010	0.0010	0.83	0.011	0.011	0.011	0.011	0.0010	0.85	0.0010	0.83	0.00	0.98	
	(0.0)	(0.1)	(0.3)	(0.1)			(0.1)	(0.1)	(0.1)	(0.0)							
PA Niacin, %	0.97	1.1	1.9	1.5	0.17	0.58	1.2	0.99	1.4	1.6	0.059	0.87	0.28	0.38	0.22	0.45	
	(2.0)	(3.0)	(7.4)	(3.3)			(3.3)	(2.5)	(3.2)	(3.3)							
PA Vitamin B6, %	3.2	3.2	2.7	2.5	0.61	0.15	3.1	3.2	2.3	2.7	0.49	0.33	0.73	0.12	0.25	0.60	
	(7.0)	(6.8)	(9.4)	(5.9)			(6.5)	(7.0)	(5.8)	(6.1)							
PA Folate, %	23	26	21	20	1.4	0.42	26	26	18	21	0.79	0.71	2.1	0.28	1.3	0.49	
	(22)	(23)	(26)	(25)			(23)	(24)	(23)	(27)							
PA Vitamin C, %	0.00	0.00	0.010	0.00	-0.0010	0.69	0.00	0.00	0.00	0.00	0.00	0.98	-0.0020	0.32	-0.0020	0.35	
	(0.0)	(0.0)	(0.1)	(0.0)			(0.0)	(0.0)	(0.0)	(0.0)							
PA Calcium, %	0.090	0.10	0.14	0.030	-0.043	0.35	0.11	0.090	0.030	0.030	-0.047	0.30	-0.038	0.40	0.010	0.37	
	(0.4)	(0.4)	(1.3)	(0.1)			(0.5)	(0.3)	(0.1)	(0.1)							
PA Vitamin B12, %	6.1	6.4	7.5	5.7	-0.79	0.71	6.6	6.2	5.3	6.2	-1.8	0.40	0.22	0.93	2.0	0.34	
	(15)	(16)	(20)	(16)			(17)	(16)	(15)	(17)							
Index Children	n=342	n=676	n=321	n=623			n=345	n=331	n=326	n=297							
Vitamin A, ìg RAE	192	165	163	177	42	0.48	161	169	191	162	62	0.41	20	0.73	-43	0.51	
	(446)	(598)	(451)	(564)			(378)	(763)	(589)	(536)							
Iron, mg	15	15	16	14	-1.6	0.27	14	15	13	15	-2.7	0.078	-0.39	0.80	2.3	0.037*	
	(11)	(12)	(13)	(10)			(12)	(12)	(9.7)	(11)							
Zinc, mg	6.0	6.1	7.0	6.3	-0.44	0.16	6.1	6.2	6.2	6.5	-0.60	0.041*	-0.27	0.50	0.34	0.28	
	(3.0)	(2.9)	(4.0)	(3.1)			(2.9)	(3.0)	(3.1)	(3.2)							
Protein, g	42	42	45	44	-0.36	0.86	41	43	41	46	-2.7	0.21	2.1	0.44	4.8	0.11	
	(28)	(22)	(25)	(24)			(22)	(23)	(21)	(26)							
Thiamin, mg	0.58	0.57	0.64	0.56	-0.059	0.19	0.56	0.57	0.52	0.60	-0.092	0.054	-0.021	0.68	0.071	0.14	
	(0.4)	(0.4)	(0.5)	(0.4)			(0.4)	(0.4)	(0.4)	(0.4)							
Riboflavin, mg	0.49	0.48	0.53	0.46	-0.027	0.57	0.48	0.48	0.42	0.49	-0.055	0.26	0.0070	0.89	0.061	0.15	
	(0.4)	(0.5)	(0.5)	(0.4)			(0.4)	(0.5)	(0.4)	(0.5)							
																	
Niacin, mg	4.7	5.0	5.8	5.1	-0.51	0.18	5.1	4.9	5.1	5.1	-0.63	0.087	-0.39	0.46	0.23	0.65	
	(3.5)	(4.6)	(4.2)	(3.3)			(5.4)	(3.6)	(3.4)	(3.2)							
Vitamin B6, mg	0.63	0.65	0.71	0.67	0.0090	0.85	0.63	0.68	0.67	0.67	0.0060	0.91	0.011	0.81	0.0050	0.91	
	(0.4)	(0.5)	(0.5)	(0.5)			(0.4)	(0.6)	(0.5)	(0.5)							
Folate, ìg	176	176	188	162	-19	0.30	171	181	154	172	-29	0.12	-8.8	0.68	20	0.18	
	(166)	(168)	(174)	(141)			(152)	(183)	(137)	(145)							
Vitamin C, mg	17	19	18	23	7.7	0.12	18	21	21	25	6.0	0.23	9.4	0.20	3.4	0.66	
	(34)	(50)	(33)	(88)			(31)	(64)	(45)	(118)							
Calcium, mg	275	267	295	260	-6.1	0.87	267	266	256	265	-11	0.78	-0.35	0.99	11	0.72	
	(331)	(325)	(427)	(310)			(295)	(354)	(305)	(315)							
Vitamin B12, ìg	0.22	0.25	0.38	0.32	0.037	0.85	0.26	0.24	0.36	0.29	0.11	0.71	-0.038	0.83	-0.15	0.60	
	(0.6)	(0.7)	(1.4)	(2.1)			(0.6)	(0.7)	(2.7)	(1.0)							
PA Thiamin, %	99	99	99	99	-0.34	0.27	100	99	100	99	0.034	0.84	-0.75	0.18	-0.79	0.18	
	(2.5)	(2.6)	(2.8)	(2.6)			(2.2)	(3.0)	(1.7)	(3.2)							
PA Riboflavin, %	17	18	6.0	6.3	0.61	0.62	18	19	6.2	6.4	0.43	0.75	0.79	0.56	0.36	0.76	
	(24)	(25)	(16)	(15)			(24)	(26)	(15)	(15)							
PA Niacin, %	20	23	32	35	0.79	0.70	23	24	35	35	1.4	0.52	0.14	0.96	-1.3	0.55	
	(21)	(24)	(29)	(30)			(24)	(24)	(29)	(31)							
PA Vitamin B6, %	87	87	59	58	-1.2	0.67	87	87	59	57	1.2	0.75	-3.8	0.23	-4.9	0.19	
	(20)	(20)	(38)	(38)			(20)	(19)	(38)	(39)							
PA Folate, %	100	100	88	88	-2.1	0.34	100	100	88	87	-0.046	0.98	-4.3	0.19	-4.2	0.21	
	(2.2)	(2.7)	(22)	(24)			(2.9)	(2.5)	(24)	(24)							
PA Vitamin C, %	44	41	16	17	0.74	0.76	41	42	16	17	0.80	0.76	0.66	0.81	-0.14	0.95	
	(39)	(38)	(26)	(29)			(37)	(39)	(28)	(29)							
PA Calcium, %	1.0	1.4	0.00	0.050	0.038	0.27	1.0	1.7	0.00	0.11	0.011	0.36	0.067	0.26	0.056	0.25	
	(5.9)	(8.2)	(0.0)	(1.1)			(6.3)	(9.8)	(0.0)	(1.6)							
PA Vitamin B12, %	12	13	11	11	-0.13	0.96	14	12	10	12	-1.2	0.64	1.1	0.70	2.3	0.33	
	(26)	(26)	(25)	(24)			(27)	(24)	(23)	(25)							
1 Descriptive values are unadjusted means with standard deviation below the mean in parenthesis. Ä is the linear regression coefficient and represents the change in the outcome in a group versus another group, as indicated in column headers. It is expressed in the same unit as the outcome or, when the unit is a percentage, it is expressed in percentage points. Stars indicate p-values of comparison of Ä to 0 value: * means p-value <0.05, which is the level of significance set for the study; ** means p-value <0.017, which is the level of significance when adjusting for multiple testing across 3 study groups using the Bonferroni method. Abbreviations used: PA, probability of adequacy
